# 776. Reducing Central Line Associated Bloodstream Infections (CLABSI) in a High-Risk Cohort of Patients by Standardizing Skin Preparation Prior to Pulmonary Artery Catheter Insertion

**DOI:** 10.1093/ofid/ofab466.973

**Published:** 2021-12-04

**Authors:** Mylinh Yun, Jay Varkey, Daniel Linehan, Elizabeth Noriega

**Affiliations:** Emory Healthcare, Atlanta, Georgia

## Abstract

**Background:**

Central line associated bloodstream infections (CLABSI) are a recognized complication of all central venous access devices including pulmonary artery catheters (PAC). At our institution, PACs are utilized frequently, often for prolonged durations, for patients with advanced heart failure in the cardiac care unit (CCU) who are awaiting heart transplant. In early summer 2018, our hospital infection prevention (IP) department detected an uptick in CLABSI attributable to the CCU. After 9 months of zero CLABSI, two CLABSIs attributable to the CCU were identified during a 3 month period from November 2017-January 2018. Four additional CLABSIs were identified between May-July 2018 prompting an investigation by IP. Review of the 9 CLABSIs attributed to the CCU from May 2018 – June 2019 led IP to prioritize improving PAC insertion practices in our cardiac catheterization lab as a mean to reducing CLABSI (see table 1).

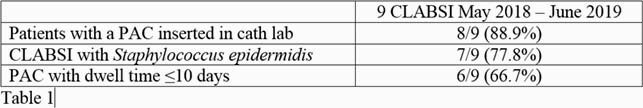

**Methods:**

IP performed 5 observations of PAC insertion in the cath lab. During the observations of skin preparation, the prep time was performed correctly 40% of the time, correct application 60% of the time and dry time 60% of the time (see table 2, Figure1). Interventions included scheduling a training day for all cath lab staff with the skin prep vendor, performing competency check-offs, and identifying super-users to train future staff. Furthermore, skin antiseptic utilization according the manufacturer's instructions for use was implemented, the coverage area for the applicator was reviewed and a chart for reference was provided.The staff was provided with posters on correct skin prep technique as a visual cue in the procedure room.

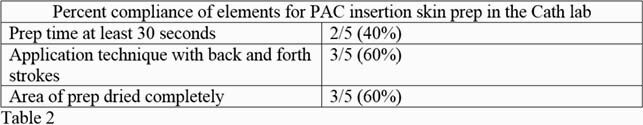

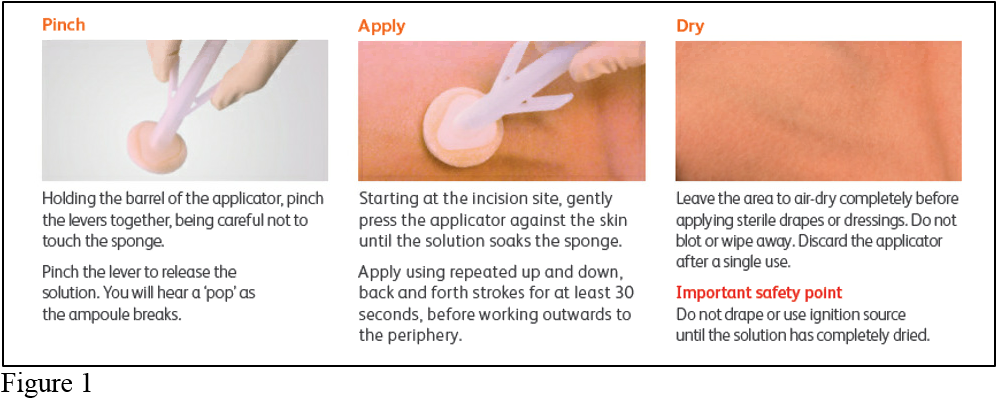

**Results:**

Since the project was implemented in September 2019, there has been 1 CLABSI identified that was possibly related to a PAC inserted in the cath lab. During this time 3 CLABSIs were identified in the CCU but were felt to be unrelated to cath lab insertion.

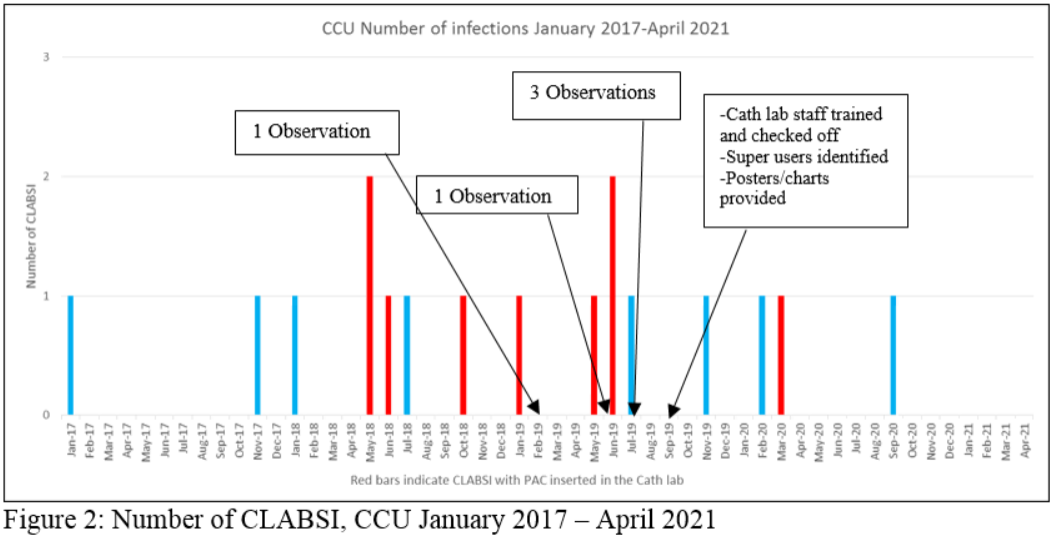

**Conclusion:**

Since the project was implemented in September 2019, there has been 1 CLABSI identified that was possibly related to a PAC inserted in the cath lab. During this time 3 CLABSIs were identified in the CCU but were felt to be unrelated to cath lab insertion.

**Disclosures:**

**All Authors**: No reported disclosures

